# *let-7-Complex* MicroRNAs Regulate Broad-Z3, Which Together with Chinmo Maintains Adult Lineage Neurons in an Immature State

**DOI:** 10.1534/g3.120.401042

**Published:** 2020-02-18

**Authors:** Yen-Chi Wu, Geetanjali Chawla, Nicholas Sokol

**Affiliations:** Department of Biology, Indiana University, Bloomington, IN 47405

**Keywords:** heterochronic, temporal identity, let-7, miR-125, Chinmo, Br-Z3

## Abstract

During *Drosophila melanogaster* metamorphosis, arrested immature neurons born during larval development differentiate into their functional adult form. This differentiation coincides with the downregulation of two zinc-finger transcription factors, Chronologically Inappropriate Morphogenesis (Chinmo) and the Z3 isoform of Broad (Br-Z3). Here, we show that *br-Z3* is regulated by two microRNAs, *let-7* and *miR-125*, that are activated at the larval-to-pupal transition and are known to also regulate *chinmo*. The *br-Z3* 3′UTR contains functional binding sites for both *let-7* and *miR-125* that confers sensitivity to both of these microRNAs, as determined by deletion analysis in reporter assays. Forced expression of *let-7* and *miR-125* miRNAs leads to early silencing of Br-Z3 and Chinmo and is associated with inappropriate neuronal sprouting and outgrowth. Similar phenotypes were observed by the combined but not separate depletion of *br-Z3* and *chinmo*. Because persistent Br-Z3 was not detected in *let-7-C* mutants, this work suggests a model in which *let-7* and *miR-125* activation at the onset of metamorphosis may act as a failsafe mechanism that ensures the coordinated silencing of both *br-Z3* and *chinmo* needed for the timely outgrowth of neurons arrested during larval development. The *let-7* and *miR-125* binding site sequences are conserved across *Drosophila* species and possibly other insects as well, suggesting that this functional relationship is evolutionarily conserved.

The heterochronic microRNAs (miRNAs) *lin-4* and *let-7* were originally identified because they ensured that cells adopted proper temporal cell fates during the development of the nematode *Caenorhabditis elegans* (*C. elegans*) (reviewed in Luhur *et al.* 2013; Faunes and Larrain 2016). As in *C. elegans*, these miRNAs display distinctive temporal expression profiles during the development of most animal species (Pasquinelli *et al.* 2000). In *Drosophila melanogaster* (*D. melanogaster*), for example, expression of *let-7* and *lin-4* ortholog *miR-125* is activated during the larval-to-pupal transition and then persists through metamorphosis and adulthood (Sempere *et al.* 2003; Chawla *et al.* 2016). This temporal control is regulated by the late larval pulse of the insect molting steroid hormone Ecdysone, which along with its nuclear receptor, Ecdysone Receptor (EcR), directly activates the *let-7-Complex* (*let-7-C*), a polycistronic locus that encodes *Drosophila let-7*, *miR-125*, and a third miRNA, *miR-100*, (Sokol *et al.* 2008; Chawla and Sokol 2012). Once activated, *let-7-C* is transcribed in cells throughout the central nervous system (CNS), where a key joint target of *let-7* and *miR-125* is Chronologically Inappropriate Morphogenesis (Chinmo) (Wu *et al.* 2012), whose post-transcriptionally controlled downregulation ensures that cells in the Mushroom Body (MB) adopt proper cell fate (Zhu *et al.* 2006; Liu *et al.* 2015). In addition to *chinmo*, *let-7* also represses *abrupt* (*ab*) allowing the timely remodeling of neuromuscular junctions (NMJs) during metamorphosis (Caygill and Johnston 2008). Chinmo and Abrupt are closely related members of the Broad-Complex/Tramtrack/Bric-a-brac (BTB) family of zinc finger (ZF) transcription factors (Spokony and Restifo 2007). Whether *let-7-C* miRNAs function in cells other than the MB and NMJ to regulate additional targets during nervous system remodeling remains unknown.

The bulk of the late larval CNS is composed of arrested immature neurons born from neural stem cells known as neuroblasts (NBs) (reviewed in Venkatasubramanian and Mann 2019). There are between 25 and 30 distinct NBs in each thoracic neuromere of the ventral nerve cord (VNC) and these NBs divide during the course of larval development to produce clusters of lineally related neurons. Because neurons in each cluster extend a primary neurite to a common destination, these clusters, or “adult lineages”, can be labeled and distinguished from one another based on cell number and neurite morphology (Truman *et al.* 2004). Terminal differentiation of these adult neurons is activated at the onset of metamorphosis, when individual neurons become distinguishable from one another by taking on unique morphologies and connections needed to generate the functional adult nervous system. While mechanisms by which sibling neurons acquire distinct identities have been uncovered in some lineages like the MB and olfactory lobe for example, less is known about this process in VNC lineages. Early and late born neurons in most VNC lineages, however, can be distinguished by the respective expression of Chinmo and the Z3 isoform of another member of the BTB-ZF family, Broad (Br), both of which persist until the onset of metamorphosis (Maurange *et al.* 2008; Zhou *et al.* 2009). While the Chinmo-to-Br-Z3 switch is associated with decreased cell size of VNC neurons, the functional roles of Chinmo and Br-Z3 as well as the mechanisms controlling their expression are less clear.

Here, we report that the 3′UTR of *br-Z3* contains functional *let-7* and *miR-125* binding sites and that, consistently, transcriptional activation of *let-7-C* coincides with downregulation of both Br-Z3 and Chinmo in the VNC. By using the mosaic analysis with a repressible cell marker (MARCM) technique (Lee *et al.* 2000b), we also find a birth-order dependent onset of *let-7-C* in many VNC adult lineages. Forced premature expression of *let-7-C* miRNAs leads to early silencing of Br-Z3 and Chinmo that is associated with premature neuronal sprouting and outgrowth. Similar phenotypes were observed by simultaneous depletion of *br-Z3* and *chinmo*, suggesting that timely onset of *let-7-C* ensures stage-specific neuronal remodeling.

## Materials and Methods

### Molecular biology and cell culture methods

*broad* cDNAs containing the *miR-125* binding site were recovered by RNA ligase-mediated rapid amplification of 5′ cDNAs (RLM-RACE) using the GeneRacer Kit (Life Technologies). A ∼4.5kb cDNA was cloned from 5 μg of total larval RNA using an oligo located 42 bp away from the *miR-125* binding site. This cDNA was sequenced completely (Figure S1), and the sequence has been deposited in GenBank (accession number MN990459). Cell culture experiments and luciferase assays were performed as described in Wu *et al.*, 2012, using psiCHECK-2 plasmids containing *br-Z3* 3′UTR (described here) or *w* 3′UTR (described in Wu *et al.* 2012).

### Transgenes and plasmids

*br-Z3* 3′UTR reporters: A 2,030 bp fragment containing the *br-Z3* 3′UTR was PCR amplified, subcloned, sequenced to confirm absence of errors, and inserted into *pP(wORF*::*MCS)* (Wu *et al.* 2012) or *psiCHECK2* (Promega) for analysis *in vivo* or in cell culture, respectively. MiRNA binding sites were deleted using Quickchange Site-Directed Mutagenesis Kit (Agilent) with oligos that removed basepairs 354-413 (*let-7* binding sites) and/or basepairs 1332-1341 (*miR-125* binding site) of the *br-Z3* 3′UTR.

*UAS-br-Z3* shRNAi transgenes: *br-Z3* siRNAs were identified by applying rules described in Fellmann *et al.* 2011 to sequence unique to the *br-Z3* open reading frame. Oligos encoding one of the identified *br-Z3* siRNAs (TTGTTGCTGTTGTTGTTCGCG) was annealed and subcloned into the EcoRI and NheI sites of pWalium20.

### Drosophila strains

Strains used included: (1) *P{w^ORF^::w^3′UTR^}* (Wu *et al.* 2012), (2) *P{w^ORF^::br-Z3^3′UTR^}* (Luhur *et al.*, 2014), (3) *P{w^ORF^::br-Z3^3′UTRΔlet-7^}*, (4) *P{w^ORF^::br-Z3^3′UTRΔmir-125^}*, (5) *P{w^ORF^::br-Z3^3′UTRΔlet-7+miR-125^}*, (6) *let-7-C^KO2^* (Wu *et al.* 2012), (7) *npr^6^* (Kiss *et al.* 1988), (8) *hs-Z3* (Bayer *et al.* 1997), (9) *{let-7-Cp^12^^.5kb^::lacZ}attP2* (Chawla and Sokol 2012), (10) *{let-7-Cp^12.5kb^::lacZ}attP40* (11) *let-7-C^Δ3miR^::optGal4* (Wu *et al.* 2012), (12) *{UAS-BrZ3^shRNAi233^}attPVK00033* (this study), (13) *FRT^40A^,* *UAS-mCD8::GFP,* *chinmo^1^* (Zhu *et al.* 2006), (14) *UAS-let-7-C* (Wu *et al.* 2012), (15) *UAS-Δlet-7-C* (Wu *et al.* 2012), (16) *UAS-chinmo-SV40,* (17) *UAS-Br-Z3-SV40,* (18) *w,* *GAL4^C155^,* *hsFLP,* *UAS-mCD8::GFP;* *FRT^40A^,* *tubP-GAL80,* (19) *w,* *GAL4^C155^,* *hsFLP,* *UAS-mRFP.LG-28a;* *FRT^40A^,* *tubP-GAL80,* and (20) *FRT^40A^.* New transgenics were generated by Rainbow Transgenic Services (California USA).

### MARCM strains and clone generation

Stocks containing either *w*, *GAL4^C155^*, *hsFLP*, *UAS-mCD8*::*GFP*; *FRT^40A^*, *tubP-GAL80* or *w*, *GAL4^C155^*, *hsFLP*, *UAS-mCD8*::*mRFP.LG^28a^*; *FRT^40A^*, *tubP-GAL80* were used to generate GFP and RFP labeled clones, respectively. Some MARCM strains also contained 2^nd^ or 3^rd^ chromosome insertions of *{let-7-Cp^12.5kb^*::*lacZ}attP2*. Flies of the appropriate strain were allowed to lay eggs for 6 hr, and then transferred to new vials. Resulting embryos were usually aged for 18 hr and then heat shocked at 38° for 30 min to generate neuroblast clones in adult lineages of 18-24 hr old animals. After heat shock, animals were reared at 25° degrees until dissection. Nervous systems were generally dissected during larval and pupal stages. Pre-wandering larvae were chosen as 3^rd^ instar larvae that remained in the food, while wandering larvae were those that had left the food. Pupae were staged by picking white prepupae every hour and then aging them on humid Petri dishes for 12, 18, and 24 hr.

### Antibody generation

To generate Br-Z3 antibodies, the 271 amino acid Z3-specific portion of the Br-Z3 protein was analyzed to find regions that maximize hydrophilicity, antigenicity, and surface probability, that contain turns, and that do not contain glycosylation sites. Based on this analysis, two peptides (residues 601-614, SLKRHFQDKHEQSD, and residues 622-636, CHRRYRTKNSLTTHK, of the BR-PA polypeptide sequence) were synthesized, coupled to KLH, and injected into rabbits (ProSci Inc, Poway, CA). Resulting polyclonal antisera were affinity-purified and found to be specific for Broad-Z3 by immunofluorescence (see Figure S3).

### Immunostaining and microscopy

Antibodies used for immunostaining included: mouse anti-nc82 (1:50, Developmental Studies Hybridoma Bank), rat-anti-Chinmo (1:500; Wu *et al.*, 2012), rabbit anti-Br-Z3 (1:250; this study), rabbit anti-GFP (1:1000; Invitrogen), chicken anti-GFP (1:500; Rockland Immunochemicals), rabbit anti-dsRed (1:500; Clontech), mouse anti-mCherry (1:500; Clontech), rabbit anti-β-gal (1:500; MP Biomedicals), chicken anti-β-gal (1:500; Abcam). Most primary antibodies were preabsorbed against fixed embryos. Secondary goat antibodies were conjugated to Alexa Fluor 488, 568 or 633 (Molecular Probes). Slides were mounted in Vectashield (Vector Labs) or dehydrated through an ethanol series and mounted in DPX. Images were collected on a Leica SP5 (Light Microscopy Imaging Center, Indiana University). Confocal stacks were merged using Leica LSM software. Samples that were directly compared were prepared under identical conditions and imaged in parallel.

### Statistical analysis

All statistical analyzes were performed using Prism (GraphPad, Version 7.0). First datasets were tested for normality using D’Agostino-Pearson test. For comparisons of two datasets, an Unpaired *t*-test was performed. Significance is indicated as follows: n, not significant; *P < 0.05; **P < 0.01; ***P < 0.001; ****P < 0.0001.

### Data availability

Strains and plasmids are available upon request. The sequence of the broad-Z3 cDNA found in Supplemental Figure 1 has been also deposited in GenBank (accession number MN990459). The authors affirm that all data necessary for confirming the conclusions of the article are present within the article, figures, and tables. Supplemental material available at figshare: https://doi.org/10.25387/g3.11858517.

## Results

### broad-Z3 3′UTR contains functional let-7 and miR-125 binding sites

**Figure 1:**
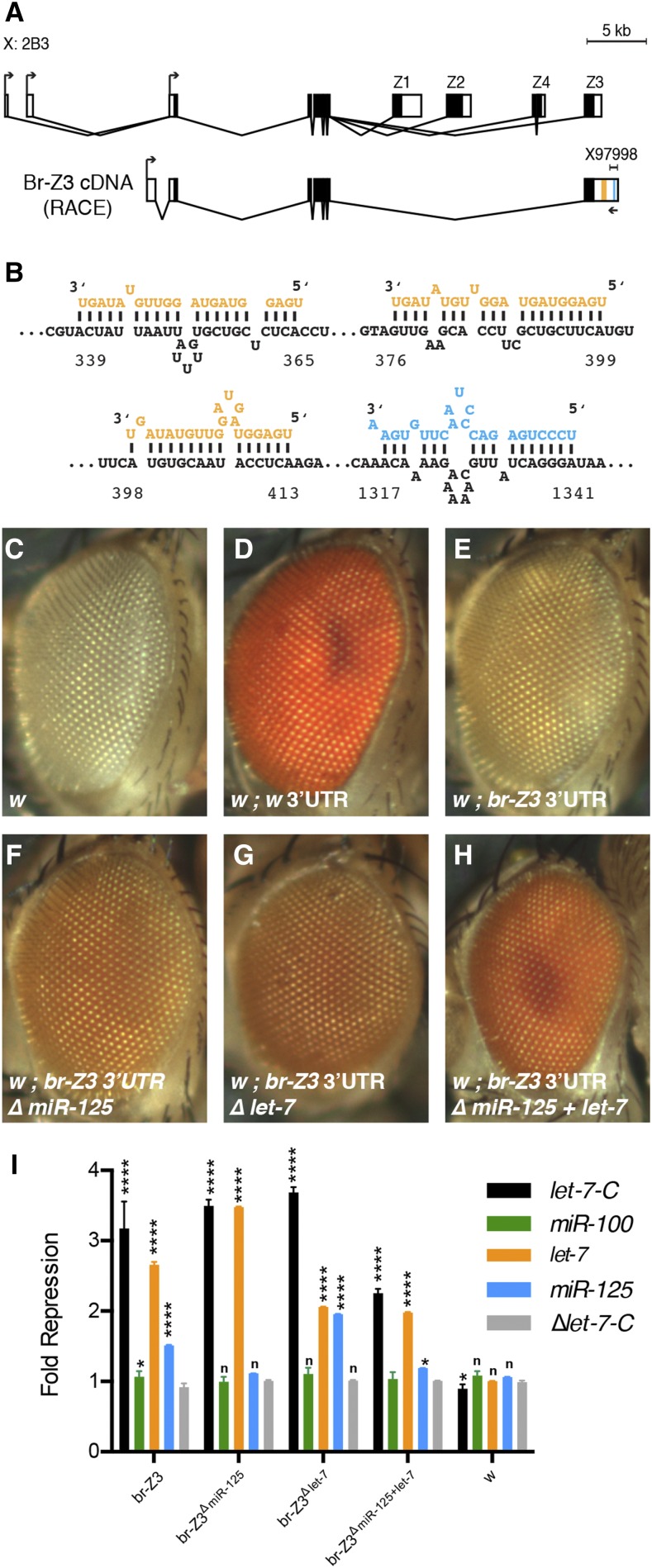
*broad-Z3 3′UTR contains functional let-7 and miR-125 binding sites*. (A) Organization of the *broad* locus (top) and RACE-identified *br-Z3* transcript (bottom). Locations of Z1-Z4 encoding exons and predicted *let-7* and *miR-125* binding sites (orange and blue lines, respectively) are shown. X97998 indicates the location of a partial *broad* cDNA predicted to contain a *miR-125* binding site originally reported in Makunin *et al.* 1996. (B) Sequences of predicted *let-7-C* binding sites in *br-Z3*. *let-7*, *miR-125*, and *br-Z3* 3′UTR sequences are shown in orange, blue, and black, respectively, and the *br-Z3* 3′UTR sequence is numbered relative to the stop codon. (C-H) Eyes of *white* mutant flies harboring reporter transgenes containing a *white* (D), *br-Z3* (E), or mutated *br-Z3* 3′UTR (F-H). (I) Fold repression of luciferase reporters containing full-length wildtype *br-Z3 *3'UTR, mutated *br-Z3* 3′UTR, or *white* 3′UTR in cell cultures in which *let-7-C* miRNAs were ectopically expressed either together or individually. ****, *P* < 0.0001; *P < 0.05; n.s. = not significant. Error bars = ± SD.

We searched available miRNA target predictions to identify additional *let-7-C* targets and found that PicTar predicted a putative *miR-125* binding site in a transcript annotated as *broad-RF* (Grün *et al.* 2005). *broad* (*br*) is a complicated locus that produces four protein isoforms (Z1-Z4) that share a common BTB domain plus one of four different zinc finger domains (Dibello *et al.* 1991; Bayer *et al.* 1996). These alternate zinc finger domains are encoded by a series of 3′ exons that also contain isoform-specific 3′UTR sequences (Figure 1A). Since the identified binding site was located in sequence from two incomplete cDNAs (X97998 and AY060896), we performed 5′ RACE to identify full-length *br* transcripts containing this predicted site using oligos located 40 nucleotides (nts) downstream of it. A 4.5 kb Broad-Z3-encoding cDNA was isolated that contained a previously unannotated first exon as well as unannotated 3′UTR sequence in the last exon (Figure 1A, Figure S1). This cDNA was consistent with RNA-seq data indicating that the last *br* exon contains an extended 3′UTR (Graveley *et al.* 2011). To determine whether the 3′UTR sequence in our novel cDNA might contain additional binding sites for *let-7-C* miRNAs, we searched it for sequences complementary to *miR-100*, *let-7* and *miR-125* using RNAhybrid (Rehmsmeier *et al.* 2004) and found three putative *let-7* binding sites along with the PicTar-predicted *miR-125* site (Figure 1B), all of which were conserved among eleven *Drosophila* species and possibly other insects as well (Figure S2). This data therefore raised the possibility that Broad-Z3 is regulated by both *miR-125* and *let-7*.

To determine whether these predicted miRNA binding sites were functional, we analyzed the *br-Z3* 3′UTR in an *in vivo* reporter system that relies on the *white* (*w*) eye pigmentation gene (Luhur *et al.*, 2014). Variation in the expression of this gene is easily detected, since it leads to gradations in eye pigment levels that vary from white to orange to bright red. For example, eye pigment levels of *w* mutant flies are fully restored with a transgene containing the *w* open reading frame (ORF) fused to its usual *w* 3′UTR (Figure 1 C, D). In contrast, eye pigment levels are only slightly increased in flies harboring the *w* open reading frame (ORF) fused to a 2 kb portion of the *br-Z3* 3′UTR identified in our cDNA (Figure 1E), suggesting that this 3′UTR mediates post-transcriptional repression of *w*. To test whether *let-7-C* miRNAs are responsible for this repression, we generated mutant versions of the *white* reporter in which either the *miR-125* site or all three *let-7* sites were deleted. Pigment levels were darker in flies harboring mutant transgenes relative to those carrying the wildtype *br* 3′UTR (Figure 1E-G), indicating that both *miR-125* and *let-7* sites were functional. Deletion of all four sites led to a further increase in pigment (Figure 1H), indicating that *miR-125* and *let-7* sites are nonredundant. Taken together, these data indicated that endogenous *let-7* and *miR-125* repressed reporter levels via binding sites in the *br-Z3* 3′UTR.

To obtain further support that the *br-Z3* 3′UTR was responsive to *let-7-C* miRNAs, we used a cell culture assay to test whether ectopic *let-7-C* miRNAs repressed expression of a luciferase reporter containing the *br-Z3* 3′UTR. Expression of *pri-let-7-C*, which encodes all three miRNAs, as well as just *let-7* or *miR-125* alone, resulted in repression of luciferase (Figure 1I). The identified *let-7* and *miR-125* binding sites in the *br-Z3* 3′UTR mediated this repression, since their deletion led to a significant reduction in the amount of repression. This analysis also indicated that the *br-Z3* 3′UTR might contain additional *let-7* sites, since the mutated reporter was still responsive to ectopic *let-7*. Nevertheless, we concluded that the identified *let-7* and *miR-125* binding sites in the *br-Z3* 3′UTR were functional.

### BTB-ZFs and let-7-C display inverse temporal expression profiles in the larval CNS

In order to investigate the *in vivo* relationship between *br-Z3* and *let-7-C* miRNAs, we generated rabbit antibodies against Br-Z3. This antisera labeled adult lineage neurons in the CNS (Figure S3). This expression profile was consistent with the reported staining patterns of both the Broad-core antisera that recognizes all four isoforms as well as previously generated mouse Broad-Z3 antisera (Mugat *et al.* 2000; Spokony and Restifo 2009; Zhou *et al.* 2009), supporting the veracity of our results. To further confirm the specificity of this staining pattern, we also stained the CNS tissue dissected from *br* mutant null allele *npr^6^* (Kiss *et al.* 1988), which affects all Broad isoforms, as well as CNS tissue from larvae in which Br-Z3 was overexpressed using a verified heatshock-inducible Z3 (*hs-Z3*) transgene (Crossgrove *et al.* 1996; Bayer *et al.* 1997). Br-Z3 staining was sharply reduced in the adult lineages of *npr^6^* mutants and elevated in *hs-Z3* CNS tissue (Figure S3). We also found that adult lineage clones expressing a *br-Z3*-specific shRNA transgene described later in the paper displayed a loss of anti-Br-Z3 staining (See Figure 5A insets). Altogether, these results indicated that our new antisera recognized Br-Z3.

**Figure 2:**
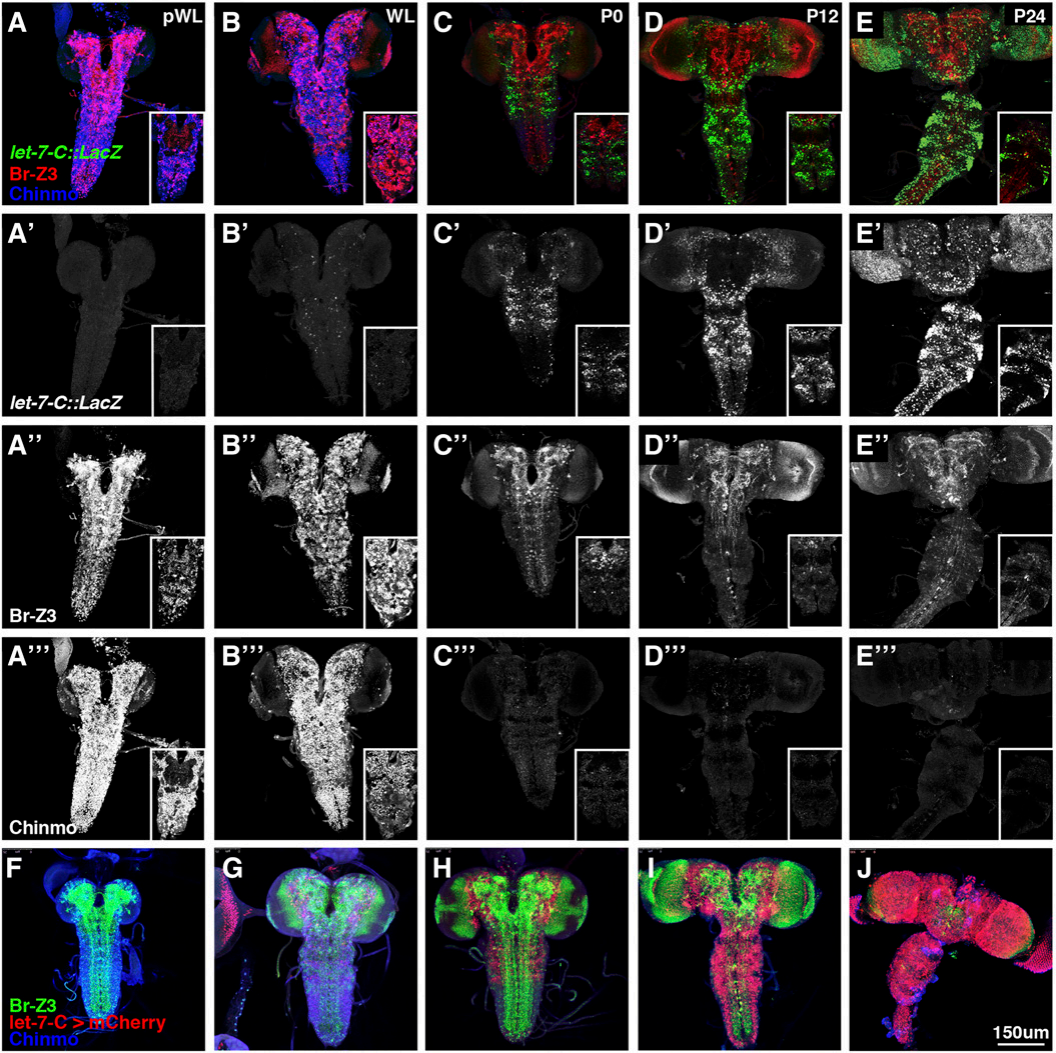
*let-7-C* expression inversely correlates with Br-Z3 and Chinmo expression in the CNS during the larval-to-pupal transition. (A-E). Dissected CNS from (A) pre-wandering larva (pWL), (B) wandering larva (WL), (C) white prepupa (P0), (D)12 hr-old pupa (12hr), and (E) 24 hr-old pupa (P24) stained for *let-7-Cp^12.5kb^::lacZ* transcriptional reporter (green in A-E, white in A’-E’), Br-Z3 (red in A-E, white in A’’-E’’) and Chinmo (blue in A-E, white in A’’’-E’’’). Insets: single confocal sections of the VNC at each timepoint. (F-J) Dissected CNS from (F) pre-wandering larva (pWL), (G) wandering larva (WL), (H) white prepupa (P0), (I)12 hr-old pupa (12hr), and (J) 24 hr-old pupa (P24) stained for *let-7-C^Δ3miR^::optGal4* > *mcherry* (red), Br-Z3 (green) and Chinmo (blue). Scale bars: A-J, 150µm.

We then used these antibodies to compare the temporal dynamics of *let-7-C* expression with Br-Z3 as well as Chinmo, a previously identified target of *let-7* and *miR-125* (Wu *et al.* 2012) throughout the entire CNS during the larval-to-pupal transition. *let-7-C* expression was monitored using a previously verified *let-7-C* transcriptional reporter, *let-7-Cp^12.5kb^*::*lacZ*, which contains a 12.5kb fragment of the first *let-7-C* intron upstream of the *LacZ* reporter gene (Chawla and Sokol 2012). Consistent with previous Northern blots of *let-7-C* miRNAs in the CNS (Chawla and Sokol 2012; Wu *et al.* 2012), the *let-7-C* transcriptional reporter was first detected at the wandering 3^rd^ larval instar stage in cells scattered throughout the central brain (CB) and VNC (Figure 2A-E). This pattern intensified and expanded by pupariation (P0), when *let-7-C* expression was most robustly detected in the thoracic segments (Figure 2C’ inset). Expression continued to expand at twelve (P12) and twenty-four hours (P24) after pupariation in the CB and VNC. Expression of *let-7-C* in the optic lobe (OL) was only first weakly detected at P12 and then more broadly at P24. As expected, the onset of *let-7-C* transcription in the CB and VNC coincided with a decrease in Br-Z3 and Chinmo. Both Br-Z3 and Chinmo were detected throughout the CB and VNC in pre-wandering and wandering third instar larvae. However, by P0, both proteins were significantly reduced and Chinmo, in particular, appeared to be almost entirely switched off. While Br-Z3 displayed a similar downregulation to Chinmo in the VNC, it persisted in the CB in the P0-P24 samples and expanded in the OL, consistent with previous reports (Riddiford *et al.* 2018), although in a pattern that was largely non-overlapping with *let-7-C*. To confirm this analysis, we compared a second *let-7-C* transcriptional reporter, *let-7-C^Δ3miR^*::*optGal4* (Wu *et al.* 2012) with Br-Z3 and Chinmo, and found a similar expression pattern (Figure 2F-J). Thus, the inverse expression of *let-7-C* and the two BTB-ZF proteins in the VNC suggested that *let-7-C* miRNAs might dampen the expression of both Chinmo and Br-Z3 during the larval-to-pupal transition.

### let-7-C is activated in a birth-order dependent pattern in adult lineages

**Figure 3:**
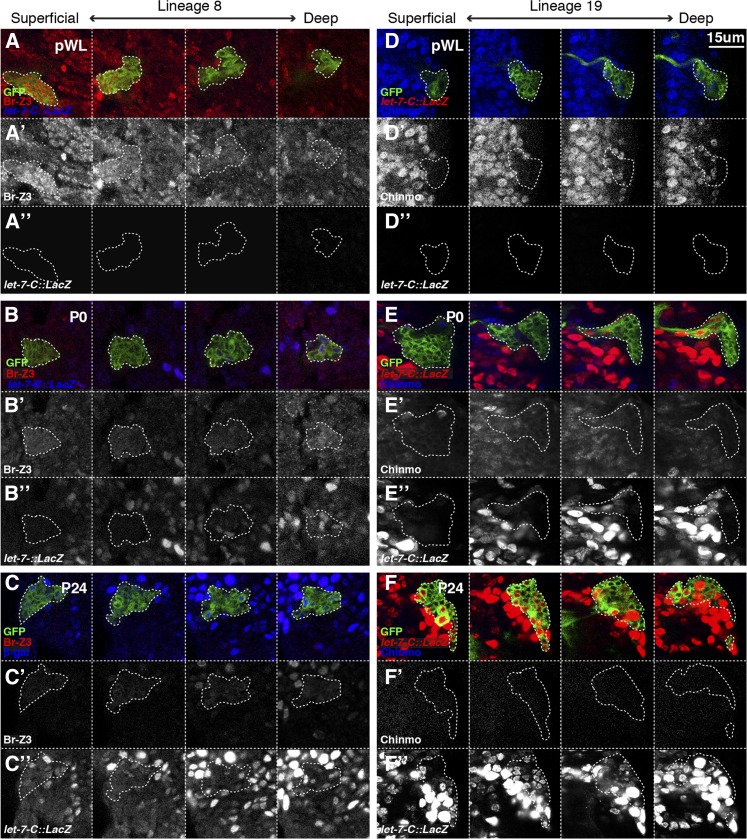
*let-7-C* expression inversely correlates with Br-Z3 and Chinmo expression in adult lineage clones during the larval-to-pupal transition. (A-C) *Elav-Gal4,* *UAS-mCD8::GFP*-labeled lineage 8 clone stained for GFP (green in A-C), Br-Z3 (red A-C, white in A’-C’), and the *let-7-Cp^12.5kb^::lacZ* transcriptional reporter (blue in A-C, white in A”-C”). Single confocal sections from the apical to basal layers show late-to-early born neurons at pWL (A), P0 (B), and P24 (C). (D-F) *Elav-Gal4*, *UAS-mCD8::GFP*-labeled lineage 19 clone stained for GFP (green in D-F), Chinmo (blue in D-F, white in D’-F’), and the *let-7-Cp^12.5kb^::lacZ* transcriptional reporter (red in D-F, white in D”-F”). Single confocal sections from the apical to basal layers show late-to-early born neurons at pWL (D), P0 (E), and P24 (F). Scale bar: A-F, 15µm.

To more finely characterize the temporal dynamics of *let-7-C*, Br-Z3, and Chinmo at the cellular level, we compared their expression in individually labeled adult lineage in the VNC before, at, and after pupariation. Adult lineages can be distinguished based on stereotypical location and neurite morphology (Truman *et al.* 2004) and, for our initial analysis, we focused in comparing *let-7-C* with Br-Z3 in lineage 8 and with Chinmo in lineage 19. Lineage 8 is a ventrolateral cluster located in the anterior half of hemimeres found in segments T1-T3, while Lineage 19 is situated dorsolaterally at the posterior border of hemimeres in segments T1-A1. In the VNC, mitotic neuroblasts and their most recently born daughters are located near the surface of the tissue whereas older born daughters are more deeply located. Consistent with the previously reported birth-order-dependent expression of Broad and Chinmo (Maurange *et al.* 2008), we found that Br-Z3+ cells were superficially located in lineage 8 whereas Chinmo+ cells were more deeply located in lineage 19 (Figure 3A’, D’). While *let-7-C*::*lacZ* was absent from labeled clones in pre-wandering larvae (Figure 3A, D), it was detected in a subset of cells in labeled lineages analyzed at pupariation (Figure 3B”, E”). These cells were located in the deep layer, indicating that *let-7-C* is first activated in older-born neurons in both lineage 8 and 19. Chinmo as well as Br-Z3 were almost entirely absent by this stage, indicating that Br-Z3 downregulation precedes *let-7-C* onset in newly born neurons and suggesting a *let-7-C* independent Br-Z3 silencing mechanism, at least in lineage 8. By P24, *let-7-C* was detected in cells ranging from the deep to superficial layers (Figure 3C”), indicating that the initial activation of let-7-C in older neurons had expanded to include more recently born neurons as well.

To determine whether *let-7-C* displayed a similar pattern of onset in other adult lineages, we quantified the number of *let-7-C*+ neurons within adult lineages located throughout the VNC at P0 and analyzed, on average, 80 clones per lineage. The majority of adult lineages (0, 1, 2, 3, 5, 6, 10, 11, 12, 13, 14, 17, 21, and 20/22) displayed a pattern similar to the one described above for lineages 8 and 19. In these cases, we found that fewer than 50% of the labeled cells were *let-7-C*+ at P0 and that these *let-7-C*+ cells were located in the deep layer, indicating that they were older-born neurons. In addition, we identified lineages that displayed one of two additional patterns. In lineages 4, 15, and 24, we found that all cells were *let-7-C*+ at P0. Lineage 15 and 24 are distinctive because they are composed entirely of motoneurons that innervate the adult leg (Baek and Mann 2009) and contain relatively few cells (10-30) compared to interneuron lineages (50-100) due to early termination of neuroblast division (Truman *et al.*, 2004). Finally, in lineages 7, 9, 16, 18, and 23, we found that greater than 50% but not all cells were *let-7-C*+ and that these cells were located in the deeper layers. Thus, with the possible exception of motoneuron lineages, we concluded that *let-7-C* is activated in a birth order dependent manner in adult lineages throughout the VNC.

### Precocious let-7-C represses Br-Z3 and Chinmo and alters neuronal morphology

**Figure 4:**
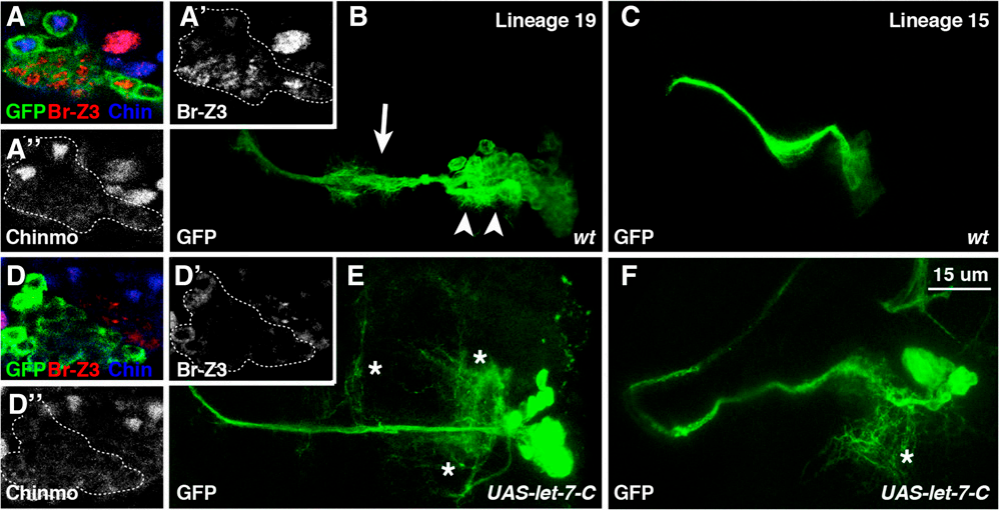
Precocious *let-7-C* expression drives premature adult lineage elaboration. (A-F) *Elav-Gal4*, *UAS-mCD8::GFP* labeled wild-type (A-C) and *UAS-let-7-C* (D-F) lineage 19 (A, B, D, E) and lineage 15 (C, F) clones stained for GFP (green in A-F), Br-Z3 (red in A, D, white in A’, D’), and Chinmo (blue in A, D, white in A”, D”). Clones were generated in newly hatched larvae and analyzed at WL. Arrow and arrowheads in B indicate contralateral and ipsilateral neurite bundles respectively. Asterisks in E and F indicate outgrowth. Scale bar: 15 μm.

Given the ability of *let-7-C* miRNAs in the MB to regulate Chinmo expression and temporal identity (Wu *et al.* 2012), we wanted to determine whether precocious *let-7-C* onset in adult lineages could lead to premature termination of Br-Z3 and Chinmo expression. We therefore compared wildtype and *UAS-let-7-C*-expressing MARCM-labeled clones induced in newly hatched larvae for Br-Z3 and Chinmo expression, focusing again on lineage 19. Within wildtype clones analyzed at the wandering third instar stage, Chinmo and Br-Z3 displayed a complementary expression pattern (Figure 4A, A’ and A’’). While *UAS-let-7-C* clones analyzed at the same timepoint contained the same number of cells as wildtype clones, they in contrast displayed a complete loss of both Chinmo and Br-Z3 proteins (compare Figure 4A and 4D). These results indicated that *let-7-C* miRNAs could repress Chinmo and Br-Z3 expression in adult lineages.

To determine the consequences of *let-7-C* expression in adult lineages, we analyzed the effect of this premature *let-7-C* expression on the neuronal morphology of specific adult lineages. During the growth of adult specific neurons, primary projections are sent out to initial targets and subsequent projections follow this same path (Truman *et al.*, 2004). The neurite is continuously maintained throughout larval development until metamorphosis, when secondary outgrowth and elaboration establishes their adult connections. By comparing neurite morphology in wildtype and *UAS-let-7-C*-expressing MARCM-labeled clones in multiple lineages, we found that premature let-7-C expression resulted in inappropriate outgrowth. For example, lineage 19 in the second thoracic hemisegment contains two primary axon bundles, one contralateral across the midline (arrow in Figure 4B) and another with ipsilateral arbor appearance (arrowheads in Figure 4B). In comparison to wildtype clones, *UAS-let-7-C* clones displayed a clear outgrowth of neurite bundles in the interstitial region of the contralateral axons as well as more complex processes on the ipsilateral arbors (asterisks in Figure 4E). These phenotypes displayed by *UAS-let-7-C*-expressing lineage 19 clones in larvae were almost identical to the morphologies displayed by wild type clones at 18 hr APF or even later timepoints (data not shown), raising the posibility that premature expression of *let-7-C* miRNAs leads to precocious neuronal remodeling.

In addition to lineage 19 phenotypes, we also characterized neurite morphology in a motoneuron-specific lineage that prematurely expressed the *UAS-let-7-C* transgene (Figure 4C). Lineage 15 exclusively contains motoneurons that project out of the CNS to the leg imaginal disc (Truman *et al.*, 2004; Baek and Mann 2009). However, in comparison to wildtype lineage 15 clones, *UAS-let-7-C* clones displayed bundles surrounded by a dense fibrous neuropil (Figure 4C, F, arrowhead). The feature of elaborated branches in motoneuron lineages with higher order is a known characteristic associated with the differentiation of adult neurons (Brierley *et al.* 2009). Thus, as with intereuron lineages, premature expression of *let-7-C* miRNAs also leads to inappropriate outgrowth of motoneuron lineages.

Since premature expression of *let-7-C* miRNAs led to inappropriate neurite outgrowth in larvae, we wondered whether loss of *let-7-C* miRNAs mutants pupae displated a delay in neuronal remodeling. To address this possibility, we generated MARCM-labeled clones in newly hatched larvae that were homozygous mutant for a *let-7-C*^KO2^, a verified *let-7-C* null allele (Wu *et al.* 2012), and examined secondary sprouting at multiple timepoints during the beginning of metamorphosis (6, 12, 18, 24 hr APF) focusing on lineage 19. The general morphology in *let-7-C* mutant clones, however, did not display an obvious phenotype; secondary outgrowth started a few hours APF and proceeded on the same pace as wild type, with elaboration and sprouting appearing as usual (data not shown). Consistent with this observation, we failed to detect persistent Br-Z3 or Chinmo expression in *let-7-C^KO2^* mutant adult lineages. Together, these observations indicate the presence of a complementary, *let-7-C* independent mechanism that promotes the timely outgrowth of adult lineages.

### Premature downregulation of Br-Z3 and Chinmo alters neuronal morphology

**Figure 5:**
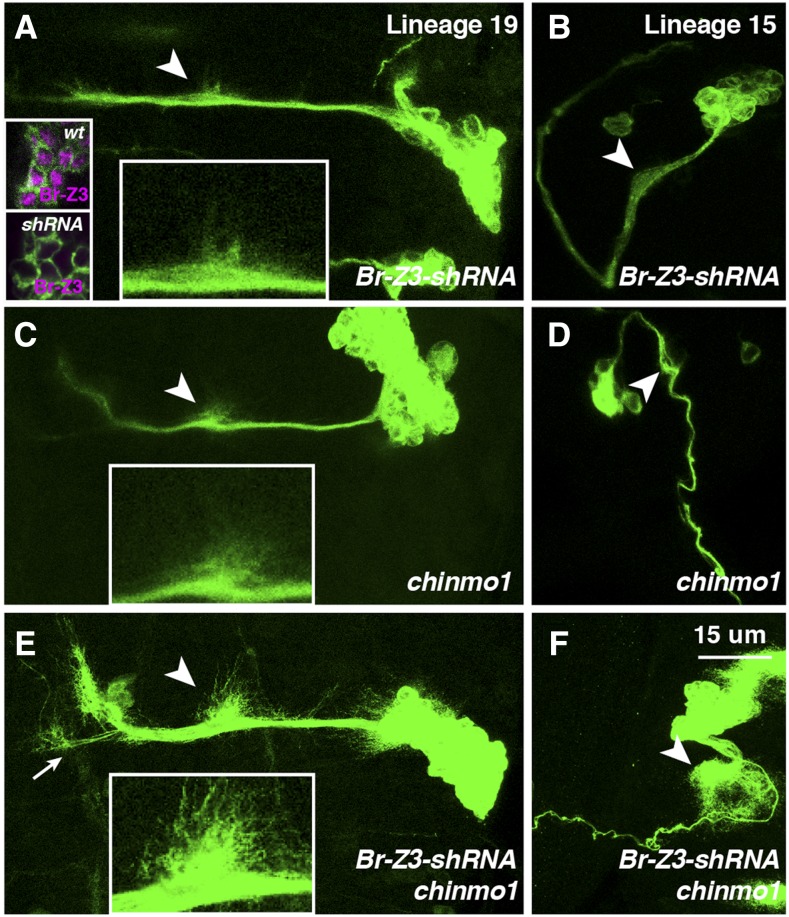
Co-depletion of Br-Z3 and Chinmo leads to premature adult lineage elaboration. (A-L) *Elav-Gal4*, *UAS-mCD8::GFP* labeled *UAS-Br-Z3-shRNA* (A-B), *chinmo^1^* (C-D), and *UAS-Br-Z3-shRNA*; *chinmo^1^* (E-F), lineage 19 (A, C, E) and lineage 15 (B, D, F) clones stained for GFP (green). Insets in A show Br-Z3 expression in wildtype (top) and *UAS-Br-Z3-shRNA* (bottom) clones, indicating both the efficacy of the shRNA as well as the specificity of the antibody. Arrowheads indicate secondary outgrowths and are enlarged in insets at bottom. Clones were generated in newly hatched larvae and analyzed at WL. Scale bar: 15 μm.

Given that *let-7-C* miRNAs can co-target both *br-Z3* and *chinmo*, the *UAS-let-7-C* phenotypes may be due to the repression of either one or both of these genes. To distinguish these possibilities, we compared the phenotypic consequences of eliminating *chinmo* and *br-Z3*, either alone or in combination, in adult lineages. The generation of double mutant homozygous clones that would be needed for this analysis is technically challenging, since *br* and *chinmo* genes are located on different chromosomes, the X and 3^rd^ respectively. To circumvent this challenge, we decided to generate *chinmo* null clones that also expressed a UAS transgene that specifically targeted the *br-Z3* transcript. We therefore designed a *UAS-br-Z3-shRNAi* transgene and tested it first by generating MARCM-labeled lineage 19 clones. Br-Z3 protein was not detectable in *UAS-br-Z3-shRNAi* clones, confirming that this transgene effectively targeted Br-Z3 as well as further indicating the specificity of the Z3 antibody (Figure 5A inset). In addition, lineage 19 and lineage 15 clones in which Br-Z3 was knocked down displayed relatively subtle outgrowth phenotypes in the neurites of adult lineages (Figure 5A, B). Similarly, homozygous *chinmo^1^* null lineage 19 and lineage 15 clones had wild-type-like larval neurite morphology (Figure 5C, D). Consistent with published reports (Maurange *et al.*, 2008), Br-Z3 and Chinmo were normally expressed in *chinmo^1^* or *UAS-br-Z3-shRNAi* clones respectively indicating that these two BTB-ZFs did not affect each other’s expression (data not shown). Since the ectopic *let-7-C* phenotypes described above were not due to depletion of either one of these targets alone, we wondered whether simultaneous elimination of both Chinmo and Br-Z3 would phenocopy the effects of *let-7-C* overexpression. We therefore generated *chinmo^1^*/*UAS-br-Z3-shRNAi* adult lineage clones at the beginning of L1. When analyzed in wandering larvae, double mutant lineage 19 clones displayed more elaborate secondary neurite outgrowth than clones of either single mutant (Figure 5E). Similarly, dual depletion in lineage 15 also caused an increased complexity of sprouting in this motoneuron lineage (Figure 5F). We also analyzed the phenotypes of dual *chinmo* and *br-Z3* depletion in additional adult lineages, and found many lineages that displayed premature outgrowth during the larval phase, including lineages 7, 11, and 24 (data not shown). The data suggested that the presence of both Chinmo and Br-Z3 prevents neurite elaboration before the pupal period. Furthermore, overexpression of *let-7-C* represses Chinmo and Br-Z3 together and leads to phenotypes similar to the combined loss of both proteins.

## Discussion

Here we provide evidence that co-transcribed miRNAs *let-7* and *miR-125* regulate Br-Z3. This conclusion is based on identification of a *br-Z3* transcript isoform that contains predicted *let-7* and *miR-125* binding sites. These sites confer *let-7*/*miR-125* sensitivity to the *br-Z3* 3′UTR as determined by the consequences of their deletion. Consistently, premature expression of *let-7* and *miR-125* eliminates the normal expression of Br-Z3 as well as a second, previously verified *let-7/miR-125* target, Chinmo, in the developing larval CNS. This forced expression of *let-7* and *miR-125* leads to inappropriate neuronal outgrowth, a phenotypic effect that is very similar to the consequences of the combined but not separate depletion of *br-Z3* and *chinmo*. Taken together, this work suggests a model in which *let-7-C* activation in the CNS during the larval-to-pupal transition ensures the coordinated silencing of both *br-Z3* and *chinmo* that is needed for the timely outgrowth during metamorphosis of neurons arrested during larval development.

The observation that Br-Z3 and Chinmo downregulation precedes the first detection of *let-7-C* transcription (see Figure 2 and 3) indicates a complementary, *let-7-C*-independent mechanism that is also responsible for Br-Z3 and Chinmo offset. Consistent with this, we failed to detect the persistence of Br-Z3 or Chinmo expression in *let-7-C* mutant adult lineages. Further supporting the absence of persistent Br-Z3 in *let-7-C* mutants, we failed to detect morphological defects in either the dendrite sensory neurons in the periphery or the lateral clock neurons in the central brain of *let-7-C* mutants, even though these two sets of neurons display axonal defects when Br-Z3 is ectopically expressed (Zhou *et al.* 2009; Scott *et al.* 2011). In addition, given the expression of *let-7-C* throughout lineages 15 and 24, we also carefully assessed the morphologies of the adult leg motoneurons in *let-7-C* mutant adults but found no obvious defects. Taken together, these observations suggest that *let-7-C* regulation of Br-Z3 is a failsafe mechanism to ensure the proper timing of neuronal remodeling when circumstances compromise *let-7-C*-independent control of Br-Z3.

The relationship between *let-7-C* miRNAs and *br-Z3* identified here extends the known connections between these miRNAs and the Ecdysone pathway and may have significance in other insects. In *Drosophila melanogaster*, the direct stimulation of *br* and *let-7-C* by Ecdysone at the onset of metamorphosis play complementary roles to activate pupal programs and repress larval ones (Bayer *et al.* 1996; Sempere *et al.* 2002; Sokol 2012; Truman and Riddiford 2019). The expression profile of Br-Z3 is therefore unusual relative to other Broad isoforms in that it transiently appears during embryogenesis in most central neurons and then persists through most of larval development in a subset of these cells while Br-Z1 and Br-Z4, in contrast, are detected only at the onset of metamorphosis (Zhou *et al.* 2009). While the function of neural Br-Z3 is not fully understood, available evidence suggests that the absence of Br-Z3 is needed for the remodeling of larval neurons to generate adult arbors. This raises the possibility that Br-Z3 might participate in maintaining neurons in an arrested, immature state, perhaps by competitively inhibiting any stochastic expression of other Br isoforms. Late larval Ecdysone expression would then lead to both the induction of Br-Z1 and -Z4 isoforms and the simultaneous elimination of Br-Z3 via let-7-C-dependent and -independent mechanisms. The conservation of the *let-7* and *miR-125* binding sites in *broad-Z3* across *Drosophila* species and possibly also in other insects suggests that the functional relationship between *br-Z3* and *let-7-C* is conserved.

Recent work has also highlighted the intimate connection between Ecdysone signaling and temporal cell fate determination pathways acting in the Drosophila nervous system. The ordered production of neurons in the mushroom body lineage, for example, was recently shown to involve a *let-7-C* dependent negative feedback loop containing Chinmo and the Ecdysone Receptor: Chinmo activates EcR, which in turn represses Chinmo by activating *let-7-C* (Marchetti and Tavosanis 2017). Similarly, EcR was also recently shown to be important for promoting the transition between early-born Chinmo-expressing neurons and late-born Broad-expressing neurons in type II neuroblasts lineages in the CNS (Syed *et al.* 2017). Our work here establishing a connection between *let-7-C* and Broad, a well-known component of the Ecdysone signaling, further extends the relationship between these two pathways.

Along with Abrupt and Chinmo, Br-Z3 is the third identified BTB-ZF target of *let-7-C* miRNAs in *Drosophila* (Caygill and Johnston 2008; Wu *et al.* 2012). These three BTB-ZFs are not only closely related to one another by sequence (Spokony and Restifo 2007), but also appear to play similar roles. All three are dosage sensitive factors that are expressed in subsets of neurons during larval development. As most clearly illustrated by genetic analysis of *abrupt* in larval dendritic arborization neurons (Li *et al.* 2004; Sugimura *et al.* 2004), these BTB-ZFs may collectively function to maintain neurons in an arrested juvenile state and are downregulated during the larval-to-pupal transition in order for neurons to achieve their mature adult form. Since BTB-ZFs are known to heterodimerize (Bonchuk *et al.* 2011), persistence of larval BTB-ZFs may inappropriately interact and interfere with the function of pupally expressed BTB-ZF cohorts. Interestingly, the temporal expression profile of *let-7-C* miRNAs is shared by isoforms of a fourth BTB-ZF, Fruitless, which are activated during metamorphosis and are closely related in sequence to Abrupt, Broad, and Chinmo (Lee *et al.* 2000a; Spokony and Restifo 2007). These observations raise the possibility that *let-7-C* miRNAs prevent the persistent expression of Abrupt, Broad, and Chinmo that could interfere with Fruitless activity, a hypothesis that future experiments can address.
